# Mortality and Hospitalizations in Intensive Dialysis: A Systematic Review and Meta-Analysis

**DOI:** 10.1177/2054358117749531

**Published:** 2018-01-10

**Authors:** Anna Mathew, Jody-Ann McLeggon, Nirav Mehta, Samuel Leung, Valerie Barta, Thomas McGinn, Gihad Nesrallah

**Affiliations:** 1 McMaster University, Hamilton, Ontario, Canada; 2 Zucker School of Medicine at Hofstra/Northwell Health, Great Neck, NY, USA; 3 Department of Nephrology, Humber River Hospital, Toronto, Ontario, Canada; 4 Faculty of Medicine, University of Toronto, Ontario, Canada

**Keywords:** intensive hemodialysis, mortality, hospitalization, meta-analysis

## Abstract

**Background:**

Survival and hospitalization are critically important outcomes considered when choosing between intensive hemodialysis (HD), conventional HD, and peritoneal dialysis (PD). However, the comparative effectiveness of these modalities is unclear.

**Objective:**

We had the following aims: (1) to compare the association of mortality and hospitalization in patients undergoing intensive HD, compared with conventional HD or PD and (2) to appraise the methodological quality of the supporting evidence.

**Data Sources:**

MEDLINE, Embase, ISI Web of Science, CENTRAL, and nephrology conference abstracts.

**Study Eligibility, Participants, and Interventions:**

We included cohort studies with comparator arm, and randomized controlled trials (RCTs) with >50% of adult patients (≥18 years) comparing any form of intensive HD (>4 sessions/wk or >5.5 h/session) with any form of chronic dialysis (PD, HD ≤4 sessions/wk or ≤5.5 h/session), that reported at least 1 predefined outcome (mortality or hospitalization).

**Methods:**

We used the GRADE approach to systematic reviews and quality appraisal. Two reviewers screened citations and full-text articles, and extracted study-level data independently, with discrepancies resolved by consensus. We pooled effect estimates of randomized and observational studies separately using generic inverse variance with random effects models, and used fixed-effects models when only 2 studies were available for pooling. Predefined subgroups for the intensive HD cohorts were classified by nocturnal versus short daily HD and home versus in-center HD.

**Results:**

Twenty-three studies with a total of 70 506 patients were included. Of the observational studies, compared with PD, intensive HD had a significantly lower mortality risk (hazard ratio [HR]: 0.67; 95% confidence interval [CI]: 0.53-0.84; *I*^2^ = 91%). Compared with conventional HD, home nocturnal (HR: 0.46; 95% CI: 0.38-0.55; *I*^2^ = 0%), in-center nocturnal (HR: 0.73; 95% CI: 0.60-0.90; *I*^2^ = 57%) and home short daily (HR: 0.54; 95% CI: 0.31-0.95; *I*^2^ = 82%) intensive regimens had lower mortality. Of the 2 RCTs assessing mortality, in-center short daily HD had lower mortality (HR: 0.54; 95% CI: 0.31-0.93), while home nocturnal HD had higher mortality (HR: 3.88; 95% CI: 1.27-11.79) in long-term observational follow-up. Hospitalization days per patient-year (mean difference: –1.98; 95% CI: –2.37 to −1.59; *I*^2^ = 6%) were lower in nocturnal compared with conventional HD. Quality of evidence was similarly low or very low in RCTs (due to imprecision) and observational studies (due to residual confounding and selection bias).

**Limitations:**

The overall quality of evidence was low or very low for critical outcomes. Outcomes such as quality of life, transplantation, and vascular access outcomes were not included in our review.

**Conclusions:**

Intensive HD regimens may be associated with reduced mortality and hospitalization compared with conventional HD or PD. As the quality of supporting evidence is low, patients who place a high value on survival must be adequately advised and counseled of risks and benefits when choosing intensive dialysis. Practice guidelines that promote shared decision-making are likely to be helpful.

## Abrégé

**Contexte:**

Au moment de choisir une modalité de dialyse pour le traitement des patients souffrant d’insuffisance rénale, le taux de survie et la durée des hospitalisations sont des critères décisionnels d’une importance cruciale. Pourtant, l’efficacité différentielle de l’hémodialyse (HD) intensive, de l’HD conventionnelle et de la dialyse péritonéale (DP) demeure à ce jour mal connue.

**Objectifs de l’étude:**

Nos objectifs allaient comme suit : 1) comparer le taux de mortalité et la durée des hospitalisations associés à chacune des modalités (HD intensive, HD conventionnelle et DP), et 2) évaluer la qualité méthodologique des données venant appuyer les résultats.

**Sources:**

Les données proviennent des bases de données en ligne MEDLINE, EMBASE et ISI Web of Science, de même que de CENTRAL et de résumés de conférence en néphrologie.

**Admissibilité à l’étude, participants et interventions:**

Ont été incluses à cette méta-analyse les études de cohorte comportant un volet comparatif et les essais contrôlés à répartition aléatoire comptant plus de 50 % de patients adultes et comparant n’importe quelle forme d’HD intensive (plus de 4 séances par semaine ou plus de 5,5 heures par séance) à n’importe quelle forme de dialyse chronique (DP ou HD à raison de 4 séances maximum par semaine ou de 5,5 heures maximum par séance). Les études retenues devaient également rapporter au moins un des deux critères décisionnels prédéfinis (mortalité et hospitalisation).

**Méthodologie:**

Nous avons employé l’approche GRADE (Grading of Recommendations Assessment, Development and Evaluation). Cette approche s’applique aux revues systématiques et à l’évaluation de la qualité des données. Deux personnes ont passé en revue des citations et des articles complets pour en extraire les données relatives à l’étude. Les divergences ont été résolues par consensus. Nous avons regroupé les différentes mesures provenant des essais à répartition aléatoire et des études observationnelles pour ensuite les analyser, de façon isolée, à l’aide de la méthode générique de l’inverse de la variance avec modèles à effet aléatoire. Pour les données où seules deux études étaient disponibles pour le regroupement des données, nous avons plutôt employé la méthode générique de l’inverse de la variance avec modèles à effet fixe. Des sous-groupes avaient été prédéfinis dans les cohortes de patients traités par HD intensive, selon le moment (de jour ou de nuit) et le lieu (en centre de dialyse ou à domicile) du traitement.

**Résultats:**

Cette méta-analyse compte 23 études totalisant 70 506 patients. Selon les études observationnelles, lorsque comparée à la DP, l’HD intensive était corrélée à un risque de mortalité significativement plus faible (HR=0,67; IC 95 0,53-0,84; I^2^=91 %). En comparaison avec l’HD conventionnelle, les schémas de traitement par HD intensive nocturne prodiguée à domicile (HR=0,46; IC 95 : 0,38-0,55; I^2^=0 %), nocturne en centre (HR=0,73; IC 95 : 0,60-0,90; I^2^=57 %) et de courte durée, de jour, à domicile (HR=0,54; IC 95 : 0,31-0,95; I^2^=82%) étaient corrélées à de plus faibles taux de mortalité. Des deux essais contrôlés à répartition aléatoire qui faisaient mention du taux de mortalité, l’HD diurne de courte durée en centre présentait le plus faible taux de mortalité (HR=0,54; IC 95 : 0,31-0,93) alors que l’HD nocturne à domicile présentait le taux de mortalité le plus élevé (HR=3,88; IC 95 : 1,27-11,79) selon les suivis observationnels faits à long terme. Le nombre de jours d’hospitalisation par année-patient (différence moyenne = -1,98 an; IC 95 : -1,59 à 2,37; I^2^=6 %) était plus faible chez les patients traités par HD intensive nocturne en comparaison avec ceux qui suivaient un traitement par la méthode conventionnelle. Dans tous les cas, la qualité des données recueillies s’est avérée faible ou très faible, qu’il s’agisse d’essais contrôlés à répartition aléatoire (en raison de l’imprécision) ou d’études observationnelles (en raison de facteurs de confusion et de biais de sélection).

**Limites de l’étude:**

Dans l’ensemble, la qualité des données recueillies pour appuyer les critères décisionnels jugés essentiels s’est avérée faible ou très faible. De plus, des éléments tels que la qualité de vie du patient, la greffe et les enjeux liés à l’accès vasculaire n’ont pas été pris en compte dans notre revue systématique.

**Conclusion:**

Le traitement de l’insuffisance rénale par HD intensive pourrait être associé à un taux de mortalité réduit et à des séjours à l’hôpital écourtés en comparaison avec les traitements par HD conventionnelle ou par DP. Cependant, en raison de la piètre qualité des données appuyant ces résultats, les patients qui accordent une grande importance à la survie devraient être adéquatement informés et conseillés sur les risques et les bienfaits offerts par l’HD intensive comme modalité de traitement. L’application de lignes directrices concernant la prise de décision conjointe en pratique clinique pourrait être pertinente.

## What was known before

Prior studies have yielded conflicting results on the effect of intensive hemodialysis on survival and hospitalization, related to differences in patient population and methodological issues such as selection bias and small sample size.

## What this adds

We systematically reviewed the available evidence on the effect of intensive hemodialysis compared with conventional hemodialysis or peritoneal dialysis on survival and hospitalization, and applied the GRADE approach to appraise the quality of evidence. We found that intensive hemodialysis regimes may be associated with reduced mortality and hospitalization, compared with conventional hemodialysis or peritoneal dialysis, but with low or very low overall quality of evidence.

## Introduction

Conventional hemodialysis (HD), comprised of 3 weekly sessions of 3- to 4-hour duration, remains the standard regimen for approximately 90% of all prevalent dialysis patients in the United States.^1^ Although survival among HD patients in the United States has improved over time, long-term survival remains comparatively poor,^2,3^ with adjusted all-cause mortality rates up to 7.9 times that of the general Medicare population.^2,3^ Approximately 1% of all US HD patients dialyze via an intensive regimen, delivered as either short daily (5-7 weekly sessions over 1.5-3 hours in duration) or nocturnal (3-7 weekly sessions over 6-8 hours in duration) treatments, in-center or at home. Intensive HD provides enhanced solute removal, and a growing body of evidence^4-9^ has suggested improvements in various physiological surrogate outcomes such as phosphate control, nutritional status, left ventricular mass, and anemia, suggesting that intensive regimens could potentially reduce the morbidity and mortality associated with HD. While conventional HD is the most common therapy, home and intensive HD therapies are becoming increasingly accessible, with more options for dialysis modalities from which patients can choose.

The comparative effects of dialysis regimens on mortality have been a major research priority for decades. While it has been argued that the dialysis comparative effectiveness research agenda should shift away from survival, and toward patient-reported outcomes,^10^ a recent international Delphi survey confirmed that both patients and health care professionals consider survival a critical outcome in dialysis treatment–related decision-making and research.^11^ Moreover, practice guidelines generally consider survival and morbidity-related events, such as hospitalization critical outcomes in formulating practice recommendations.^12^

It is well recognized that studies reporting survival outcomes with intensive HD—both randomized trials and observational designs—have yielded conflicting results due to various factors, including differences in study populations and other methodological issues.^13^ For clinicians seeking to engage patients in shared decision-making around modality choice, these seemingly disparate findings are barriers to truly informed discussions of benefits and harms.

We therefore undertook this systematic review and meta-analysis of mortality and hospitalization comparing intensive HD with other dialytic therapies. Our primary objective was to use formal methodological quality appraisal methods to determine which bodies of evidence should be used to inform decision-making through future practice guidelines and patient decision-aids addressing modality selection.

## Materials and Methods

See Appendix A for detailed methods. This article was prepared in accordance with PRISMA (Preferred Reporting Items for Systematic Reviews and Meta-analyses) guidelines.^14^ An experienced health information specialist developed the search strategies using terms to identify studies of intensive dialysis (see Appendix B for sample search strategy). We included cohort studies with comparator arm, and randomized controlled trials (RCTs) with >50% of adult patients (≥18 years) comparing any form of intensive HD (>4 sessions/wk or >5.5 h/session) with any form of chronic dialysis (peritoneal dialysis, HD ≤4 sessions/wk or ≤ 5.5 h/session), that reported at least 1 predefined outcome (mortality or hospitalization). We excluded studies of hemodiafiltration, hemofiltration, continuous renal replacement therapy, acute kidney injury, and pre-post studies with no separate patient cohort as a comparator arm. To reduce era effects, we excluded studies published before 2000. Two reviewers independently screened citations, evaluated the eligibility of each full-text article using prepiloted eligibility forms, and resolved discrepancies by consensus.

The 2 outcomes assessed were mortality and hospitalization, all-cause or cause-specific. Hospitalization was defined by either the admission rate or the number of days in hospital (per patient-year). We did not collect individual patient-level data. Two reviewers independently extracted study-level data from included studies using custom-made data extraction forms. For each outcome of interest, we extracted the unadjusted effect estimate, any adjusted effect estimates with factors included in the adjusted model, and methodological factors relevant to the quality appraisal. Disagreements in data collection were resolved by consensus.

### Methodological Quality Appraisal

We applied the GRADE quality appraisal criteria summarized in GRADE evidence profile tables, which include risk of bias,^15^ indirectness,^16^ inconsistency,^17^ imprecision,^18^ and publication bias.^19^ For RCTs, risk of bias was assessed using criteria proposed by the Cochrane Collaboration.^20^ For observational studies, we used the modified Newcastle-Ottawa criteria proposed by the CLARITY Group.^21^

### Data Synthesis

We planned to compute pooled effect estimates of randomized and observational studies separately, and used the *I*2 statistic to quantify heterogeneity. We used mean differences to pool the continuous outcomes of hospitalization days/patient-year and hospitalization rates/patient-year, and used hazard ratios to pool the dichotomous outcome of mortality. We used a random effects model to account for within- and between-study heterogeneity when there were more than 2 pooled studies, and a fixed model when there were 2 studies.^22^ All statistical analyses were conducted using Review Manager (RevMan) Version 5.3 Copenhagen: The Nordic Cochrane Centre, The Cochrane Collaboration, 2014.

## Results

### Study Characteristics

**Figure 1.**
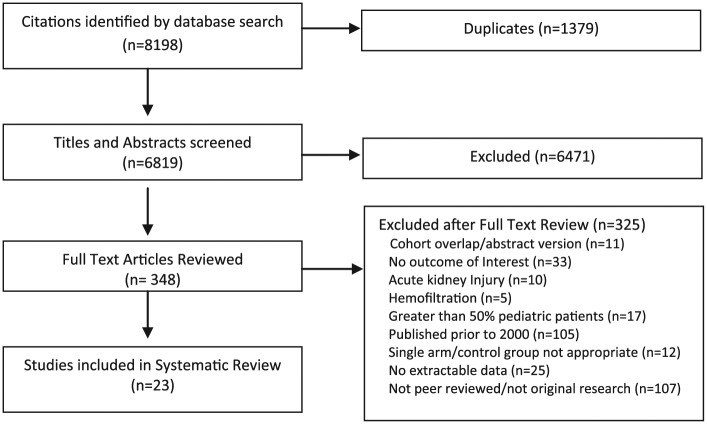
Study flow diagram.

Our search yielded 8198 citations. After excluding 1379 duplicates, 6819 citations were screened and 348 were reviewed in full-text. Twenty-three articles fulfilled all eligibility criteria and were included in the final review^23-45^ (Figure 1), with a total of 70 506 reported patients (45 370 on conventional HD, 9582 on PD, and 15 444 on intensive dialysis). Three of the 23 included studies were RCTs,^25,28,42^ and the remaining 20 were observational cohort studies. Follow-up ranged from 1 to 23 years. Study population mean age ranged from 40.9 to 55.8 years in the intensive HD group, and from 40.9 to 62.4 years in the comparator group (conventional HD or PD) (Table 1). Definitions for intensive dialysis varied by study, with 8 studies of frequent short daily HD (ranging from 5 to 6 days per week) and 15 studies of long nocturnal HD (ranging from 5.0 to 10 hours per day).

**Table 1.** Baseline Characteristics of Included Studies.

Author	Year	Country	Study duration, y	Study design	Funding source	Sample size		Mean age, y (SD)		Dialysis regimen			Home vs in-center		% Prevalent patients
						Intensive	Control	Intensive	Control	Intensive		Control	Intensive	Control	
										h/day (mean)	d/wk (mean)				
**Mortality studies**															
Intensive HD vs conventional HD															
Johansen^a^ NHD	2009	USA	10	Pros. Obs.	NIH	94	940	47.0 (16.3)	46.7 (17.5)	7.5 ± 0.82	5.7 ± 0.44	3×/wk	Home	In-center	100
Johansen^a^ SDHD	2009	USA	10	Pros. Obs.	NIH	43	430	40.9 (17.3)	40.9 (19.1)	2.9 ± 0.59	5.4 ± 0.50	3×/wk	Home	In-center	100
Lacson	2012	USA	2	Pros. Obs	Fresenius Medical Care	746	2062	52.8 (13.4)	54.1 (14.4)	7.85 ± .52	3 ± (NR)	3×/wk	In-center	In-center	100
Chertow	2015	USA	4	RCT	NIH, NIDDK, CMS, DaVita, Dialysis Clinics, Fresenius Medical Care, Renal Advantage, Renal Research Institute, Satellite Healthcare	125	120	48.9 (13.6)	52.0 (14.1)	2.5 ± 0.33	5.17 ± 1.11	2.88 ± 0.39 sessions per week	In-center	In-center	100
Marshall NHD	2013	Australia, New Zealand	10	Pros. Obs	Abbott Australasia Pty Ltd, Roche Products NZ Ltd, Novartis NZ Ltd & Fresenius Medical Care–Asia-Pacific Pty Ltd	714	3608	51.1 (NR)	58.2 (NR)	≥5.0^g^	≥3×/wk^d^	3×/wk^e^	Home	In-center	NR
Weinhandl	2012	USA	3	Pros. Obs.	NxStage Medical Inc	1873	9365	52.2 (14.8)	53.2 (14.7)	NR	5-6 sessions per week	3×/wk	Home	In-center	100
Rocco	2015	USA	3.7	RCT	NIH, NIDDK, CMS	45	42	51.7 (14.4)	54.0 (12.9)	≥6	5.06 (0.80)	2.91 (0.21)	Home	Home	100
Nesrallah	2012	Canada, France, USA	10	Ret. Obs.	Baxter Healthcare Corporation, Gambro R&D, Fresenius Medical Care, and the Canadian Institutes for Health Research, Heart and Stroke Foundation of Ontario	338	1388	50.8 (12.4)	52.3 (12.4)	7.35 (0.87)	4.8 (1.1)	3×/wk	Home	In-center	100
Ok^a^	2011	Turkey	1	Pros. Obs	European Nephrology and Dialysis Institution (ENDI, Germany)	247	247	45.2 (13.9)	45.8 (12.9)	7.5 (0.33)	3.9 (0.11)	3×/wk	In-center	In-center	100
Von Gersdorff	2010	Germany	3	Pros. Obs.	NR	494	494	NR	NR	>7	NR	3×/wk	NR	NR	100
Kjellstrand Italy	2008	Italy	23	Pros. Obs.	NR	165	NR	51 (15)	NR	2.3 (0.5)	NR	3×/wk	Home 46%	In-center	91
Kjellstrand USA	2008	USA	23	Pros. Obs.	NR	169	NR	55 (15)	NR		NR	3×/wk	Home 70%	In-center	
Kjellstrand France/UK	2008	France, UK	23	Pros. Obs.	NR	81	NR	45 (14)	NR		NR	3×/wk	Home 88%	In-center	
Blagg	2006	USA	2	Pros. Obs.	NR	117	NR	55.5 (NR)	NR	2-3.5	≥5×/wk	3×/wk	Home 83.8%	NR	100
Lockridge	2011	USA	12	Pros. Obs.	NR	87	NR	52 (15)	NR	7 (1)	NR	3×/wk	Home	In-center	NR
Suri	2013	Canada, USA, France	10	Pros. Obs.	CIHR	318	575	55.8 (18)	56 (13)	2.7 (0.7)	5.8 (0.5)	3×/wk	In-center	In-center	100
Intensive HD vs PD															
Marshall^b^ PD	2013	Australia, New Zealand	10	Pros. Obs.	Abbott Australasia Pty Ltd, Roche Products NZ Ltd, Novartis NZ Ltd & Fresenius Medical Care–Asia-Pacific Pty Ltd	714	2649	51.1 (NR)	60.4 (NR)	≥5.0^b^	≥3×/wk^c^	PD	−-	−-	NR
Nesrallah	2016	USA	12	Ret. Obs.		2668	2668	51.3 (14.3)	51.4 (14.1)	1.5-3 h	5-7	PD	Home	−-	100
Weinhandl^a^	2016	USA	3	Ret. Obs.		4201	4201	53.8 (14.9)	54.6 (15.0)	NR	5-6	PD	Home	−-	100
**Hospitalization studies**															
Intensive HD vs conventional HD															
Van Eps	2010	Australia	3	Pros. Obs.	NHMRC	63	172	51.7 (12.9)	58.3 (15.5)	6-9	3.5-5	3×/wk	Home	In-center	100
Lindsay NHD	2003	Canada	2.5	Pros. Obs.	NR	12	17	44.2 (6.4)	48.8 (11.9)	6-8	5-6	3×/wk	Home	In-center	100
Bergman	2008	Canada	2	Pros. Obs.	Heart and Stroke Foundation, Physician Services Incorporated Foundation	32	42	43 (2)	44 (2)	8-10	5-6	3×/wk	Home	In-center	100
Zimbudzi	2014	Australia	1	Ret. Obs.	NR	25	25	53.6 (NR)	47.4 (NR)	8^g^	4	3×/wk	Home	In- center	100
Lacson	2010	USA	1	Pre-post Obs.	Fresenius Medical Care	655	15 334	51.2 (12.7) *P* < .0001	62.4 (15.0)	7.85 ± 0.48	3	3×/wk	In-center	In-center	100
Weinhandl^b^	2015	USA	5	Ret. Obs.	NxStage Medical Inc	2084	10 420	54.0 (NR)	54.3 (NR)	NR	5-6	3×/wk	Home	In-center	100
Culleton	2007	Canada	2	RCT	Kidney Foundation of Canada	26	25	55.1 (12.4)	53.1 (13.4)	6	5-6	3×/week	Home	In-center	100
Intensive HD vs PD															
Kumar	2008	USA	5	Pros. Obs.	NR	22	64	52^f^	54^f^	2.45 (0.3)	5.4 (0.5)	PD	Home	−-	23

*Note.* HD = hemodialysis; PD = peritoneal dialysis; NHD = nocturnal hemodialysis; SDHD = short daily hemodialysis; NR = not reported. Pros. Obs = Prospective Observational Ret. Obs = Retrospective Observationa NHMRC = National Health and Mental Research Council NIH = National Institutes of Health NIDDK = National Institute for Diabetes and Digestive Diseases CMS = Centers for Medicare and Medicaid Services CIHR = Canadian Institutes for Health Research

a Hospitalization outcomes also reported.

b Most contemporary reported cohort.

c In 95% of sample.

d In 99% of sample.

e In 94% of sample.

f Median.

g In 61% of sample.

### Results of Individual Studies

Effect estimates for mortality and hospitalization in individual studies are described in Tables 2 and 3. Factors included in adjustment analysis varied across studies (Table 4).

**Table 2.** Mortality Event Rates From Individual Studies.

Author/year	Sample size		Event rate, per patient-year		Unadjusted effect estimate		Adjusted effect estimate	
	Intensive	Control	Intensive	Control	HR (95% CI)	*P* value	HR (95% CI)	*P* value
**Intensive HD vs conventional HD**								
Mortality								
Johansen NHD 2009	94	940	0.074	0.154	NR	NR	0.36 (0.22-0.61)	.00001
Johansen SDHD 2009	43	430	0.091	0.139	NR	NR	0.64 (0.31-1.31)	.22
Lacson 2012	746	2062	142^a^	557^a^	0.69 (0.58-0.84)	<.001	0.75 (0.61-0.91)	.004
Marshall NHD 2013	714	3608	NR	NR	0.4 (0.33-0.49)	<.05	0.46 (0.37-0.56)	<.05
Weinhandl 2012	1873	9365	0.110	0.127	NR	NR	0.87 (0.78-0.97)	.01
Nesrallah 2012	338	1388	0.061	0.105	0.39 (0.29-0.52)	NR	0.55 (0.34-0.87)	.01
Ok 2011	247	247	0.0177	0.0623	0.28 (0.09-0.85)	.02	0.68 (0.1-0.98)	.04
Von Gersdorff 2010	494	494	0.031	0.066	NR	NR	0.75 (NR)	<.03
Kjellstrand-Italy 2008	165	NR	0.066	NR	NR	NR	0.34 (0.20-0.54)	<.001
Kjellstrand-USA 2008	169	NR	0.143	NR	NR	NR		
Kjellstrand-France/UK 2008	81	NR	0.048	NR	NR	NR		
Blagg 2006	117	NR	0.076	NR	NR	NR	0.39 (0.19-0.51)	<.005
Lockridge 2011	87	NR	0.0453	NR	NR	NR	0.30 (NR)	(NR)
Suri 2013	318	575	0.156	0.109	1.6 (1.1-2.3)	.023	1.3 (1.02-1.7)	0.034
Hospital admission rate (admissions per patient-year)								
Ok 2011	247	247	0.65	2.26	NR	NR	NR	NR
Lindsay-NHD 2003	12	17	0.95 ± 1	0.93 ± 1.2	NR	NR	NR	NR
Van Eps 2010	63	172	2.0 (1.7-2.3)	1.75 (1.54-1.98)	NR	NR	NR	NR
Bergman 2008	32	42	0.21 ± 0.07	0.49 ± 0.12	NR	NR	NR	NR
Zimbudzi 2014	25	25	0.72	0.72	NR	NR	NR	NR
Lacson 2010	655	15 334	1.26	1.74	NR	NR	NR	NR
Weinhandl 2015	2084	10 420	1.78	1.69	NR	NR	1.03 (0.99-1.08)	NR
Johansen NHD 2009	94	940	1.1	0.9	NR	NR	NR	NR
Johansen SDHD 2009	43	430	0.6	0.7	NR	NR	NR	NR
Hospitalization day rate (hospital days per patient-year)								
Lindsay-NHD 2003	12	17	4.8 ± 7	4.54 ± 6.5	NR	NR	NR	NR
Van Eps 2010	63	172	9.2 (8.6-9.9)	11.61 (11.06-12.19)	NR	NR	NR	NR
Bergman 2008	32	42	1.49 ± 0.66	3.37 ± 1.03	NR	NR	NR	NR
Zimbudzi 2014	25	25	2.8 (NR)	3.4 (NR)	NR	NR	NR	NR
Lacson 2010	655	15 334	9.6 (NR)	13.5 (NR)	NR	NR	NR	NR
Weinhandl 2015	2084	10 420	9.64	9.91	NR	NR	1.01 (0.94-1.07)	NR
Johansen NHD 2009	94	940	5.8 (NR)	5.6 (NR)	NR	NR	NR	NR
Johansen SDHD 2009	43	430	3.1 (NR)	3.1 (NR)	NR	NR	NR	NR
**Intensive HD vs PD**								
Mortality								
Weinhandl 2016	4201	4201	0.121	0.151	NR	NR	0.8 (0.73-0.87)	<.001
Nesrallah 2016	2668	2668	0.127	0.167	0.84 (0.82-0.86)	<.001	0.75 (0.68-0.82)	<.001
Marshall PD 2013	714	2649	NR	NR	0.35 (0.26-0.43)	<.05	0.45 (0.37-0.56)	<.05
Hospital admission rate (admissions per patient-year)								
Kumar 2008	22	64	0.68 (NR)	0.76 (NR)	0.78	.5	0.98	.9
Weinhandl 2016	4201	4201	1.74	1.99	NR	NR	0.92 (0.89-0.95)	NR
Hospitalization day rate (hospital days per patient-year)								
Kumar 2008	22	64	3.3	5.6	0.37	.06	1.23	.8
Weinhandl 2016	4201	4201	10.27	12.67		NR	0.81 (0.75-0.87)	NR

*Note.* CI = confidence interval; HR = hazards ratio; NHD = nocturnal hemodialysis; SDHD = short daily hemodialysis; NR = not reported.

a Reported as absolute number of events.

**Table 3.** Mortality Event Rates From Randomized Controlled Trials.

Author/year	Sample size		Event rate, per patient-year		Unadjusted effect estimate		Adjusted effect estimate	
	Intensive	Control	Intensive	Control	HR (95% CI)	*P* value	HR (95% CI)	*P* value
**Intensive HD vs conventional HD**								
Mortality								
Chertow 2015	125	120	20^a^	34^a^	NR	NR	0.54 (0.31-0.93)	NR
Rocco 2015	45	42	14^a^	5^a^	NR	NR	3.88 (1.27-11.79)	.01
Hospital admission rate^b^								
Culleton 2007	26	25	0.62 (0.24-1.00)^a^	0.84 (0.18-1.50)^a^	NR	NR	NR	NR

*Note.* CI = confidence interval; HR = hazards ratio; NR = not reported.

a Reported as absolute number of events.

b Mean rate per patient from baseline to study exit (study duration August 2004 to December 2006).

**Table 4.** Factors Adjusted and Not Adjusted for in Multivariable Analysis and/or Study Design.

First Author	Year	Age	BMI	Comorbid conditions*	Country	Diabetes	Dialysis vintage	Dry weight	Education level	ESRD Cause	ESRD duration	ESRD start date	Ethnicity	Gender	GFR	HD dose	HD session length	Hemoglobin	Hospitalization	Medicaid status	Primary diagnosis	Race	Smoking	Dialysis modality	Time on HD	Vascular access	LVM	Urine volume
Johansen	2009	√	√	√		√				√				√					√	√		√				√		
Lacson	2010	√	√			√	√							√												√		
Lacson	2012	√	√				√			√																		
Nesrallah	2012	√				√								√								√						
OK	2010	√				√								√			√								√			
Suri	2012	√		√	√	√	√	√						√												√		
Von Gersdorff	2010	√				√								√														
Kjellstrand	2008	√				√								√							√							
Blagg	2006	√								√				√								√						
Lindsay	2003	√		√		√								√										√		√		
Lockridge	2011	√		√			√		√					√			√					√						
Van Eps	2010	√	√	√										√			√						√					
Bergman^a^	2008	√		√		√								√														
Weinhandl	2014	√	√	√						√	√			√					√			√						
Marshall	2013	√	√	√		√				√			√	√									√					
Nesrallah	2016	√		√						√		√		√				√				√						
Weinhandl	2016	√	√	√						√		√		√	√			√										
Kumar	2008	√					√							√								√		√				
Weinhandl	2012	√	√	√						√	√			√					√			√						
Zimbudzi^b^	2014																											

*Note.* BMI = body mass index; ESRD = end-stage renal disease; GFR = glomerular filtration rate; HD = hemodialysis; LVM = left ventricular mass.

a Additionally adjusted for Charlson comorbidity index; cardiovascular-related, myocardial infarction; congestive heart failure; peripheral vascular disease; cerebrovascular disease; hyperparathyroidism; and cancer.

b Did not adjust for any factors.

* Additionally adjusted for Charlson Comorbidity Index, cardiovascular-related, myocardial infarction, congestive heart failure, peripheral vascular disease, cerebrovascular disease, hyperparathyroidism or cancer.

- Mortality: Thirteen studies^23-35^ examined mortality in intensive HD compared with conventional HD (2 RCTs and 11 observational studies). The 2 RCTs^25,28^ were long-term follow-up studies from the Frequent Hemodialysis Trials group, analyzed using intention-to-treat principles, but with inconsistent continuation of the randomization intervention. In a follow-up study to the Frequent Hemodialysis Network (FHN) short daily trial over a median of 3.6 years, Chertow et al described the relative mortality hazard for daily versus conventional HD as 0.54 (95% confidence interval [CI]: 0.31-0.93). Similarly, in a follow-up to the FHN nocturnal trial over a median of 3.7 years, Rocco et al described the relative mortality hazard for follow-up for nocturnal versus conventional HD as 3.88 (95% CI: 1.27-11.79). Of the remaining 11 observational studies, the adjusted hazard ratio (HR) for intensive HD compared with conventional HD ranged from 0.36 (95% CI: 0.22-0.61) to 0.87 (95% CI: 0.78-0.97).

Three observational studies examined mortality in intensive HD compared with PD.^26,43,45^ The adjusted HR for intensive HD compared with PD varied from 0.45 (95% CI: 0.37-0.56) to 0.8 (95% CI: 0.73-0.87).

ii. Hospitalization: One RCT^42^ reported adverse events of mean hospitalizations per patient from baseline to study exit in both the nocturnal (0.62; 95% CI: 0.24-1.00) and conventional HD groups (0.84; 95% CI: 0.18-1.50) (Tables 2 and 3).

Ten observational studies reported hospitalization rates. Eight compared intensive HD with conventional HD,^23,30,36-41^and 2 compared intensive HD with PD^43,44^ (Tables 2 and 3). Only 3 of these studies also reported an unadjusted and/or adjusted relative treatment effect estimate comparing intensive HD with PD or conventional HD.^41,43,44^

### Synthesis of Results

**Figure 2.**
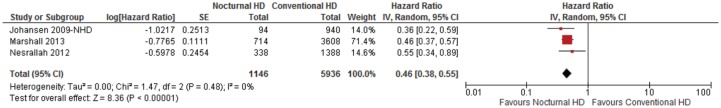
Comparative risk of mortality in nocturnal home HD versus conventional HD.

**Figure 3.**
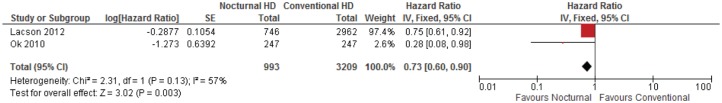
Comparative risk of mortality in nocturnal in-center HD versus conventional HD.

**Figure 4.**
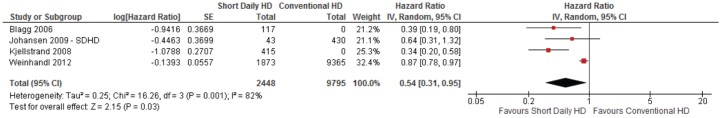
Comparative risk of mortality in short daily home HD versus conventional HD.

**Figure 5.**
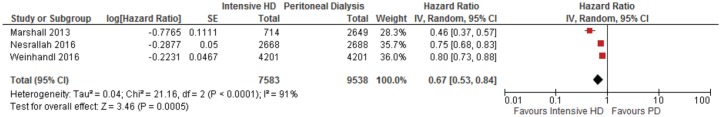
Comparative risk of mortality in intensive HD versus PD.

**Figure 6.**
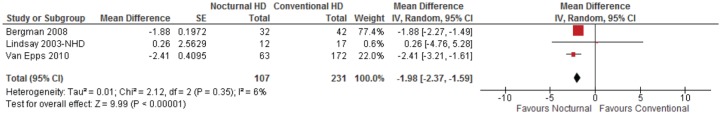
Comparative mean difference in hospitalization days/patient-year for nocturnal home HD versus conventional HD.

**Figure 7.**
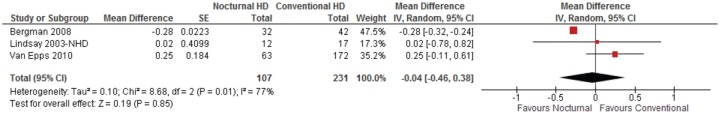
Comparative mean difference in hospital admission rate/patient-year for nocturnal home HD versus conventional HD.

Due to incomplete data reporting, only 13 of the 23 studies in this systematic review were included in the meta-analysis^23,26,27,29,30,32,33,36-38,43,45,46^ (Figures 2-7 and Tables 5-8).

**Table 5.** GRADE Evidence Profile Table: Effects of Nocturnal Home HD Compared With Conventional HD in Patients on Chronic HD.

Quality assessment							No. of patients		Effect		Quality	Importance
No. of studies	Study design	Risk of bias	Inconsistency	Indirectness	Imprecision	Other considerations	Nocturnal home hemodialysis	Conventional hemodialysis	Relative (95% CI)	Absolute (95% CI)		
All-cause mortality in observational studies												
3	Observational studies	Serious^a^	Not serious	Not serious	Not serious	Strong association	−/1146	−/5936	HR 0.46 (0.38-0.55)	—^b^	⊕⊕⚪⚪ Low	Critical
All-cause mortality in randomized trials												
1	Randomized trials	Serious^c^	Not serious	Not serious	Serious^d^	None	14/45 (31.1%)	5/42 (11.9%)	HR 3.88 (1.27-11.79)	269 more per 1000 (from 30 more to 657 more)	⊕⊕⚪⚪ Low	Critical
Mean hospital days in observational studies (assessed with hospital days per patient-year)												
3	Observational studies	Serious^a^	Not serious	Not serious	Serious^e^	None	107	231	—	MD 1.98 lower (2.37 lower to 1.59 lower)	⊕⚪⚪⚪ Very low	Critical
Mean hospitalization rate in observational studies (assessed with hospitalizations per patient-year)												
3	Observational studies	Serious^a^	Serious^f^	Not serious	Serious^g^	None	107	231	—	MD 0.04 lower (0.46 lower to 0.38 higher)	⊕⚪⚪⚪ Very low	Critical
Mean hospitalization rate in randomized trials (assessed with hospitalizations per patient-year)												
1	Randomized trials	Serious^h^	Not serious	Not serious	Serious^d^	None	27	25	—	MD 0.22 lower	⊕⊕⚪⚪ Low	Critical

*Note.* CI = confidence interval; HR = hazard ratio; MD = mean difference.

a Risk of bias due to incomplete adjustment for prognostic factors in statistical analysis.

b Absolute event counts not provided, precluding estimation of absolute event rates.

c Extremely low control group event rate suggests uneven baseline prognosis between treatment groups.

d Low event rates and small overall sample size reduce precision for this outcome; optimal information size criterion not met.

e Small sample size; observed effect may be due to random error.

f *I*^2^ = 77% for pooled effect estimate, possibly due to unexplained heterogeneity in study population and study design.

g CI overlaps, no effect.

h Lack of blinding may have biased hospitalization practices and adjudication of hospitalization events.

**Table 6.** GRADE Evidence Profile Table: Effects of Nocturnal In-Center HD Compared With Conventional HD in Patients on Chronic HD.

Quality assessment							No. of patients		Effect		Quality	Importance
No. of studies	Study design	Risk of bias	Inconsistency	Indirectness	Imprecision	Other considerations	Nocturnal in center HD	conventional HD	Relative (95% CI)	Absolute (95% CI)		
Mortality												
2	Observational studies	Serious^a^	Serious^b^	Not serious	Not serious	None	−/993	−/3209	HR 0.73 (0.60 to 0.90)	**—** ^c^	⊕⚪⚪⚪ Very low	Critical

*Note.* HD = hemodialysis; CI = confidence interval; HR = hazard ratio;

a Some concern for incomplete adjustment for prognostic factors in statistical analysis.

b *I*2 = 57% for pooled effect estimate, could not exclude heterogeneity due to study design.

c Absolute event counts not provided, precluding estimation of absolute event rates.

**Table 7.** GRADE Evidence Profile Table: Effects of Short Daily Home HD Compared With Conventional HD in Patients on Chronic HD.^a^

Quality assessment							No. of patients		Effect		Quality	Importance
No. of studies	Study design	Risk of bias	Inconsistency	Indirectness	Imprecision	Other considerations	Short daily home HD	Conventional HD	Relative (95% CI)	Absolute (95% CI)		
Mortality												
4	Observational studies	Serious^a^	Serious^b^	Not serious	Not serious	None	−/2448	−/9795	HR 0.54 (0.31 to 0.95)	**—** ^c^	⊕⚪⚪⚪⚪Very low	Critical

*Note.* Only 1 study compared short daily in-center HD with conventional HD, precluding pooling. HD = hemodialysis; CI = confidence interval; HR = hazard ratio.

a Concerns that selection of exposed and unexposed from different population, and concern for residual confounding.

b *I*^2^ = 82% for pooled effect estimate, possibly due to unexplained heterogeneity in study design.

c Absolute event counts not provided, precluding estimation of absolute event rates.

**Table 8.** GRADE Evidence Profile Table: Effects of Intensive HD Compared With PD in Patients on Chronic HD.

Quality assessment							No. of patients		Effect		Quality	Importance
No. of studies	Study design	Risk of bias	Inconsistency	Indirectness	Imprecision	Other considerations	Intensive HD	PD	Relative (95% CI)	Absolute (95% CI)		
Mortality												
3	Observational studies	Serious^a^	Serious^b^	Not serious	Not serious	None	−/7583	−/9538	HR 0.67 (0.53 to 0.84)	**—** ^c^	⊕⚪⚪⚪ Very low	Critical

*Note.* HD = hemodialysis; PD = peritoneal dialysis; CI = confidence interval; HR = hazard ratio.

a Concern for lack of matching on prognostic factors and adjustment in statistical analysis.

b *I*^2^ = 91% for pooled effect estimate, with unexplained heterogeneity possibly due to study design and population.

c Absolute event counts not provided, precluding estimation of absolute event rates.

- Nocturnal HD versus conventional HD: Three observational studies^23,26,29^ reported risk of all-cause mortality in nocturnal home HD compared with conventional HD, with a pooled hazard ratio of 0.46 (95% CI: 0.38-0.55; *I*^2^ = 0%), favoring nocturnal home HD over conventional HD. Two observational studies^30,46^ reported risk of all-cause mortality in nocturnal in-center HD compared with conventional HD, and favored nocturnal in-center HD (HR: 0.73; 95% CI: 0.60-0.90; *I*^2^ = 57%). Only 1 RCT^28^ by Rocco et al reported mortality in this patient group, precluding pooling.

Three studies^36-38^ reported mean hospitalization days per patient-year in nocturnal home HD compared with conventional HD, with a pooled mean difference of −1.98 (95% CI: –2.37 to −1.59; *I*^2^ = 6%) favoring nocturnal HD. The mean hospital admission rate per patient-year favored nocturnal home HD, with a pooled mean difference of −0.04 (95% CI: –0.46 to 0.38; *I*^2^ = 77%).

ii. Short Daily HD versus conventional HD: Four studies^23,27,32,33^ reported risk of all-cause mortality in short daily home HD compared with conventional HD, and favored short daily home HD (HR: 0.54; 95% CI: 0.31-0.95; *I*^2^ = 82%).

One observational study^35^ by Suri et al and one RCT^25^ by Chertow et al compared short daily, in-center HD with conventional HD, precluding pooling of estimates for this predefined group.

iii. Intensive HD versus PD: Three studies^26,43,45^reported risk of all-cause mortality in intensive HD compared with PD (2 examined nocturnal home HD, and 1 examined short daily, home HD). Pooled HR was 0.67 (95% CI: 0.53-0.84; *I*^2^ = 57%) favoring intensive HD over PD.

The remainder of studies in predefined patient groups did not report adequate data such as measures of dispersion, precluding pooling.

### Methodological Quality

Tables 5 to 8 summarize the quality appraisal by predefined patient groups on an outcome-by-outcome basis. RCTs assessed outcomes of mortality and hospitalization rate in nocturnal home HD. Quality of evidence for the RCTs assessing mortality was low (imprecision). Quality of evidence for the RCT assessing hospitalization rate was also low (imprecision and risk of bias due to lack of blinding). For observational studies, risk of bias was serious in all pooled estimates. Concern for risk of bias was due to incomplete adjustment for all important prognostic factors and selection of exposed and unexposed cohorts from different populations (Appendix C). Inconsistency (due to heterogeneity from study design, study population characteristics, treatment indication, or unexplained heterogeneity) and imprecision (due to small sample size or CIs overlapping no effect) also affected the quality of most estimates. Small numbers of included studies in any predefined patient group precluded meaningful analysis of publication bias by funnel plots. The overall quality of evidence was low or very low for critical outcomes.

## Discussion

To our knowledge, this is the first systematic review and meta-analysis of mortality and hospitalization in intensive HD compared with conventional HD and PD. Compared with conventional HD, nocturnal home HD, nocturnal in-center HD, and short daily home HD were all significantly associated with decreased mortality. Intensive HD was also significantly associated with decreased mortality when compared with PD. With respect to hospitalization outcomes, nocturnal home HD was significantly associated with decreased rate of hospitalization days per year, but had no appreciable association with the rate of hospital admissions per year. The overall quality of evidence for these outcomes was similarly low across observational studies (primarily due to residual confounding and selection bias) and RCTs (primarily related to imprecision due to small study populations and low event rates) for a given modality comparison.

Among the studies reporting outcomes with nocturnal home HD, one RCT by Rocco et al^28^ reported higher mortality in patients on nocturnal home HD versus conventional HD. In contrast, our pooled analysis of observational studies found reduced mortality with home nocturnal HD. Reasons for this discrepancy may include the following: (1) The RCT was not powered to detect differences in mortality alone, and observed differences in patient survival could be explained by chance alone; (2) RCT conventional HD participants had a very low death rate of 0.032 events per patient-year, 5-fold lower compared with HD patients in the US Renal Data System,^47^ thus increasing the risk of type I error; (3) frequent modality changes over long-term follow-up precluded attributing causality to the baseline dialysis regimen—the as-treated analysis of the FHN nocturnal cohorts using the prior 6-month average exposure in fact found no significant difference in long-term survival^28^; (4) in the observational studies, patients who selected home nocturnal HD represent a healthy population with lower mortality risk, with residual confounding remaining despite statistical adjustment; and (5) loss of residual renal function in the predominantly incident nocturnal HD patients of the RCT (median dialysis vintage 0.9 years) may have contributed to the observed increased mortality.^48,49^ The observational studies in our pooled analysis did not report residual kidney function, but included prevalent patients who had likely lost most residual function at the time of cohort entry. Patients on nocturnal dialysis in the reports by Johansen et al^23^ and Nesrallah et al^29^ had a mean time on dialysis of 5 to 6 years at enrolment. Marshall et al^26^ included only incident conventional HD patients, while intensive HD patients were all prevalent patients. Loss of residual kidney function in the conventional HD group may have contributed to their observed increased mortality.

Among the studies reporting on short daily HD patients treated in-center, we identified one observational study and one RCT. Using international registry data and a matched cohort design, Suri et al^35^ reported higher mortality with in-center short daily HD compared with conventional HD (very low quality of evidence due to risk of bias from incomplete risk adjustment). Conversely, in the long-term follow-up study of FHN daily trial participants, Chertow et al^25^ reported lower mortality for in-center short daily HD patients (moderate quality of evidence—rated down one level for imprecision). However, patients in the study by Suri et al were older, had more comorbidities, had a high overall mortality rate, and were typically prescribed daily HD as a “salvage” therapy.^35^ Their higher death rate compared with matched controls may have been due to incomplete risk adjustment for disease severity, frailty, and other factors. Conversely, patients in the FHN daily study were healthier and younger and clinical trial participants with an unusually low death rate of only 4% in the first year.^25^ These studies therefore inform clearly different clinical effectiveness questions.

Our pooled results also indicated that intensive HD was associated with a lower mortality than PD (very low quality evidence due to risk of bias and inconsistency). All 3 studies included in the meta-analysis compared a home intensive HD regimen with PD, and used advanced modeling and matching techniques to account for measured between-group case-mix differences. Unmeasured potent prognostic factors such as self-efficacy or functional ability may have resulted in some residual confounding favoring home intensive HD.^50,51^ The available data did now enable a subgroup analysis evaluating the effects of a PD-first approach among patients who later switch to intensive home HD.

We identified only 1 RCT^42^ examining hospitalization in intensive HD, which precluded pooling. Our meta-analysis of studies examining hospitalization outcomes found that intensive HD was associated with a lower number of hospitalization days per patient-year. This is in line with findings by Ting et al^8^ (excluded for no comparator group), where 42 patients who were converted from conventional HD to short daily HD had a 34.4% reduction in hospitalization days. For intensive HD patients who dialyze at home, greater self-efficacy may have facilitated earlier discharge from hospital.

It is important to note that the intervention of intensive HD itself may confer complications beyond those included in our review. Increased access frequency has been associated with complications including need for thrombectomy and surgical revision.^52^ Alternate needling methods, such as buttonhole cannulation, may be associated with increased risk of infection.^53^ Long hours of nocturnal HD may lead to electrolyte imbalances (hypokalemia, hypophosphatemia), and fluid removal associated hypotension with organ ischemia.^54^ More frequent or long hours of exposure of blood to the dialyzer membrane may be associated with increased inflammation^55^ and decreased survival.^56,57^

Strengths of our study include the use of rigorous systematic review and quality appraisal methods, resulting in evidence summaries that are usable by a range of audiences. Our study’s limitations are primarily those of the included studies as described in our quality appraisal. Additional potential limitations include the following: (1) Our a priori definitions of eligible intensive dialysis prescriptions may have resulted in exclusion of some studies; (2) we identified some variability in dialysis technology, including the use of low-flow dialysate systems, which may have introduced clinical heterogeneity in our meta-analyses; and (3) our findings should not be extrapolated to patients outside of the inclusion criteria of the RCTs and observational studies in this review, which generally include nonpregnant, maintenance HD patients without mental incapacity, medical contraindications to intensive dialysis, or short lifespan (eg, less than 6 months). In addition, the FHN Short Daily study^25^ excluded patients with residual kidney function of greater than 3 mL/min per 35 L. Finally, we did not study quality of life, transplantation, and vascular access outcomes, and cannot issue general guidance regarding modality choice and these critical outcomes.

Based on our findings, we recommend several future avenues of research and work. First, clinical practice guidelines and decision-aids addressing dialysis modality selection with an emphasis on shared-decision making are needed. While strong recommendations based on high-quality evidence are desirable, guidelines can be most useful when there is less certainty surrounding treatment effects. When confidence in treatment effect measures is low, guideline statements will typically be qualified or “conditional,” and provide direction not on a specific treatment option, but rather on how clinicians should engage patients in shared decision-making, including which values and preferences to elicit when considering a pair of treatment alternatives.^58^ Second, our review findings challenge the notion that all dialysis modalities provide similar outcomes. Currently, the only modality selection guideline published to date endorses a “modality-neutral” approach, in which patients are advised to focus on preferences rather than outcomes.^59^ However, our findings suggest that some fully informed and highly motivated patients may consider more intensive regimens. Patients who place a very high value on survival may choose an intensive HD regimen despite the increased effort and despite the uncertainty in the published evidence. Thus, comparative effectiveness research of dialysis modalities is needed to aid in reducing uncertainty around candidate treatment alternatives, and obtaining truly informed consent. Third, international standards for patient decision-aids have been established and the inclusion of up-to-date quality-appraised evidence summaries of dialysis modality selection in these knowledge products is considered essential to truly informed patient choice.^60^ Finally, studies evaluating the effects of intensive dialysis for patients with specific clinical indications (frailty, severe heart disease, restoring fertility, improving obstetrical outcomes) would be of significant value.

## Conclusion

Home and intensive HD therapies continue to proliferate globally, calling on more clinicians to engage patients in discussing increasingly complex treatment decisions. We found the quality of supporting evidence is low, and thus, patients who place a high value on survival must be adequately advised and counseled of risks and benefits when choosing intensive dialysis. Survival is but one among several critical outcomes that patients must weigh against their other needs, values, and preferences. Moving toward more transparent and evidence-informed decision making seems not only timely but essential.

## Appendix A

### Detailed Methods

We registered our protocol with PROSPERO (registration number: CRD42014005270).^15^ We applied the GRADE approach to systematic reviews and quality appraisal,^16-18^ and prepared this article in accordance with PRISMA (Preferred Reporting Items for Systematic Reviews and Meta-analyses) guidelines.^19^

#### Literature search

We searched MEDLINE (1966 to March 2016 including in-process and other nonindexed citations), Embase (1980 to March 2016), ISI Web of Science (1976 to March 2016, including conference abstracts), and CENTRAL. An experienced health information specialist developed the search strategies using terms to identify studies of intensive dialysis. The final search strategies (Appendix A) included search terms “hemodialysis,” “peritoneal dialysis,” “intensive,” “frequent,” and others, with index headings and search terms adapted for each database. We did not restrict our search by language, country, outcome, or journal. We also compiled citations from hand-searching bibliographies of identified articles and previous reviews, and related articles in PubMed and Google Scholar. In addition, we searched conference abstracts from 2010 to March 2016 for ongoing or completed studies. We downloaded all citations into Endnote X7 for de-duplication and then imported into DistillerSR20, an online collaborative systematic review software tool used for screening, calibration, and data extraction.

#### Study selection

We included all cohort studies with parallel arm control groups, comparator arm, and randomized controlled trails (RCTs) with >50% adult patients (≥18 years) comparing any form of intensive hemodialysis (HD) (>4 sessions/wk or >5.5 h/session) with any form of chronic dialysis (peritoneal dialysis, HD ≤4 sessions/wk or ≤5.5 h/session). Included studies reported at least 1 of our predefined outcomes of interest (mortality or hospitalization), and needed to include at least 10 adult patients on intensive dialysis and 10 adult patients on any other form of chronic dialysis. When multiple publications described overlapping patient cohorts, we reviewed all publications but based on our review on the most recent and comprehensive report. When both the abstract and full-text manuscript were available for the same patient cohort, we included only the full-text study. We excluded studies of hemodiafiltration, hemofiltration, continuous renal replacement therapy, acute kidney injury, and pre–post studies with no separate patient cohort as a comparator arm. We excluded letters, commentaries, editorials, and any other article with no original data. To reduce era effects, we excluded studies published before 2000.

Two reviewers (A.M. and S.L.) independently screened each citation using pretested forms. We retrieved full-text articles for any citation considered potentially relevant by either reviewer. Two reviewers (A.M. and N.M.) independently evaluated the eligibility of each full-text article using prepiloted eligibility forms, and resolved discrepancies by consensus.

#### Outcomes

The 2 outcomes assessed were mortality and hospitalization, all-cause or cause-specific. Hospitalization was defined by either the admission rate or the number of days in hospital (per patient-year).

#### Data collection

We did not collect individual patient-level data. Two reviewers (N.M. and J-.A.M.) independently extracted study-level data from included studies using custom-made data extraction forms. Data items included study design, methods, patient characteristics, definition of intensive dialysis and comparator group, and outcomes. For each outcome of interest, we extracted the unadjusted effect estimate, any adjusted effect estimates with factors included in the adjusted model, and methodological factors relevant to the quality appraisal. Disagreements in data collection were resolved by consensus, with involvement by a third reviewer (A.M.), if needed.

#### Methodological quality appraisal

We applied the GRADE quality appraisal criteria, which include risk of bias,^21^ indirectness,^22^ inconsistency,^23^ imprecision,^24^ and publication bias.^25^ We planned to assess publication bias through visual inspection of funnel plots. For RCTs, risk of bias was assessed using criteria proposed by the Cochrane Collaboration.^26^ For observational studies, we used the modified Newcastle-Ottawa criteria proposed by the CLARITY Group20. We summarized meta-analysis and quality appraisal findings in GRADE evidence profile tables. We assessed the quality of evidence separately for each treatment comparison and outcome using 4 GRADE quality ratings (very low, low, moderate, and high). Pooled effect estimates based on randomized trials begin with a high-quality rating, and can be rated down for serious methodological limitations in any of the 5 quality domains. Pooled estimates based on observational studies begin with a low-quality rating, and can be further rated down based on the 5 quality domains, and can also be rated up for a large magnitude of effect, dose-response gradient, or antagonistic bias (when all plausible residual confounding would result in an overestimate of effect).^27^

#### Data synthesis

We quantified interreviewer agreement using Cohen’s kappa coefficient. To reduce anticipated heterogeneity due to patient characteristics and/or study design, our a priori planned synthesis of results by groups was as follows: (1) RCT data were reported separately from observational studies; (2) short daily HD (>4 times per week) data were analyzed separately from nocturnal (>5.5 hours per session) data for conventional HD comparisons; (3) home and in-center patients were reported separately for conventional HD comparisons; (4) all intensive HD estimates were pooled together for comparisons with PD; and (5) when available, adjusted estimates from individual studies were used for pooling. We pooled risk estimates using the generic inverse variance method, and we planned to compute pooled effect estimates of randomized and observational studies separately. We used the *I*^2^ statistic to quantify the magnitude of heterogeneity. We used mean differences to pool the continuous outcomes of hospitalization days/patient-year and hospitalization rates/patient-year, and used hazard ratios to pool the dichotomous outcome of mortality. We used a random effects model to account for within- and between-study heterogeneity when there were more than 2 pooled studies, and a fixed model when there were 2 studies.^28^ All statistical analyses were conducted using Review Manager (RevMan) Version 5.3 Copenhagen: The Nordic Cochrane Centre, The Cochrane Collaboration, 2014.

## Appendix B

### Sample Search Strategy

1. Hemodialysis, Home/
2. (short daily adj2 (dialysis or hemodialysis or haemodialysis)).tw.
3. (long daily adj2 (dialysis or hemodialysis or haemodialysis)).tw.
4. (home adj2 (dialysis or hemodialysis or haemodialysis)).tw.
5. ((nocturnal or night$) adj2 (dialysis or hemodialysis or haemodialysis)).tw.
6. (intensive adj2 (dialysis or hemodialysis or haemodialysis)).tw.
7. (daily adj (haemodialysis or hemodialysis)).tw.
8. or/1-7
9. remove duplicates from 8

## Appendix C

**Table:** Risk of Bias Assessment for Studies Included in Meta-Analysis.

Author	Year	Q1	Q2	Q3	Q4	Q5	Q6	Q7	Q8
Johansen	2009	Definitely yes	Definitely yes	Definitely yes	Mostly yes	Probably yes	Definitely yes	Definitely yes	Definitely yes
Lacson	2010	Probably yes	Definitely yes	Definitely yes	Mostly yes	Probably yes	Definitely yes	Definitely yes	Definitely yes
Lacson	2012	Definitely yes	Definitely yes	Definitely yes	Mostly yes	Probably yes	Definitely yes	Definitely yes	Probably yes
Nesrallah	2012	Probably no	Definitely yes	Definitely yes	Mostly yes	Probably yes	Definitely yes	Definitely yes	Definitely yes
OK	2010	Definitely yes	Definitely yes	Definitely yes	Mostly yes	Probably yes	Definitely yes	Definitely yes	Probably yes
Suri	2012	Probably yes	Definitely yes	Definitely yes	Mostly yes	Probably yes	Definitely yes	Definitely yes	Probably yes
Von Gersdorff	2010	Definitely yes	Definitely yes	Definitely yes	Mostly no	Probably yes	Definitely yes	Definitely yes	Probably yes
Kjellstrand	2008	Probably no	Definitely yes	Definitely yes	Mostly yes	Mostly yes	Definitely yes	Definitely yes	Probably yes
Blagg	2006	Probably no	Definitely yes	Definitely yes	Mostly no	Probably no	Probably yes	Probably yes	Definitely no
Lindsay	2003	Definitely no	Definitely yes	Definitely yes	Mostly no	Mostly no	Definitely yes	Probably yes	Probably yes
Lockridge	2011	Definitely no	Definitely yes	Definitely yes	Mostly yes	Probably yes	Definitely yes	Definitely yes	Probably yes
Van Epps	2010	Probably no	Probably yes	Definitely yes	Mostly yes	Mostly yes	Definitely yes	Probably yes	Probably yes
Bergman	2008	Probably no	Definitely yes	Definitely yes	Mostly yes	Probably yes	Definitely yes	Definitely yes	Probably yes
Weinhandl	2014	Definitely yes	Definitely yes	Definitely yes	Mostly yes	Probably yes	Definitely yes	Definitely yes	Probably yes
Weinhandl	2012	Definitely yes	Definitely yes	Definitely yes	Mostly yes	Probably yes	Definitely yes	Definitely yes	Probably yes
Marshall	2013	Probably no	Definitely yes	Definitely yes	Mostly yes	Probably yes	Definitely yes	Definitely yes	Probably yes
Nesrallah	2016	Probably yes	Definitely yes	Probably yes	Mostly yes	Probably yes	Definitely yes	Definitely yes	Probably yes
Weinhandl	2016	Probably yes	Definitely yes	Definitely yes	Mostly yes	Probably yes	Definitely yes	Definitely yes	Probably yes
Kumar	2008	Definitely yes	Definitely yes	Definitely yes	Mostly yes	Probably yes	Definitely yes	Definitely yes	Probably yes
Zimbudzi	2014	Definitely yes	Definitely yes	Definitely yes	Mostly no	Definitely no	Definitely yes	Probably yes	Probably no

### Tool to Assess Risk of Bias in Cohort Studies

1. Was selection of exposed and nonexposed cohorts drawn from the same population?
2. Can we be confident in the assessment of exposure?
3. Can we be confident that the outcome of interest was not present at start of study
4. Did the study match exposed and unexposed for all variables that are associated with the outcome of interest or did the statistical analysis adjust for these prognostic variables?
5. Can we be confident in the assessment of the presence or absence of prognostic factors?
6. Can we be confident in the assessment of outcome?
7. Was the follow-up of cohorts adequate?
8. Were co-interventions similar between groups?

## References

[1] United States Renal Data System. 2017 USRDS annual data report: Epidemiology of kidney disease in the United States. National Institutes of Health, National Institute of Diabetes and Digestive and Kidney Diseases, Bethesda, MD, 2017

[2] van Walraven C Manuel DG Knoll G. Survival trends in ESRD patients compared with the general population in the United States. Am J Kidney Dis. 2014;63:491-499. 24210591 10.1053/j.ajkd.2013.09.011

[3] ESRD Mortality. *Chapter 6: Mortality* (Vol. 2, pp. 400-403). In: United States Renal Data System (ed.). USRDS Coordinating Center; 2016.

[4] Jardine M Zuo L Gray N et al Impact of extended weekly hemodialysis hours on quality of life and clinical outcomes: the ACTIVE dialysis multinational trial. J Am Soc Nephrol. 2014;25:B2.

[5] Ayus JC Mizani MR Achinger SG Thadhani R Go AS Lee S. Effects of short daily versus conventional hemodialysis on left ventricular hypertrophy and inflammatory markers: a prospective, controlled study. J Am Soc Nephrol. 2005;16:2778-2788. 16033855 10.1681/ASN.2005040392

[6] Woods JD Port FK Orzol S et al Clinical and biochemical correlates of starting “daily” hemodialysis. Kidney Int. 1999;55:2467-2476. 10354296 10.1046/j.1523-1755.1999.00493.x

[7] Kooistra MP Vos J Koomans HA Vos PF. Daily home haemodialysis in The Netherlands: effects on metabolic control, haemodynamics, and quality of life. Nephrol Dial Transplant. 1998;13:2853-2860. 9829490 10.1093/ndt/13.11.2853

[8] Ting GO Kjellstrand C Freitas T Carrie BJ Zarghamee S. Long-term study of high-comorbidity ESRD patients converted from conventional to short daily hemodialysis. Am J Kidney Dis. 2003;42:1020-1035. 14582046 10.1016/j.ajkd.2003.07.020

[9] Galland R Traeger J Arkouche W Delawari E Fouque D. Short daily hemodialysis and nutritional status. Am J Kidney Dis. 2001;37:S95-S98. 11158870 10.1053/ajkd.2001.20758

[10] Lee MB Bargman JM. Survival by dialysis modality—who cares? Clin J Am Soc Nephrol. 2016;11:1083-1087. 26912541 10.2215/CJN.13261215 PMC4891763

[11] Evangelidis N Tong A Manns B et al Developing a set of core outcomes for trials in hemodialysis: an International Delphi Survey. Am J Kidney Dis. 2017;70:464-475. 28238554 10.1053/j.ajkd.2016.11.029

[12] Guyatt GH Oxman AD Kunz R et al GRADE guidelines: 2. Framing the question and deciding on important outcomes. J Clin Epidemiol. 2011;64:395-400. 21194891 10.1016/j.jclinepi.2010.09.012

[13] Miller AJ Perl J Tennankore KK. Survival comparisons of intensive vs. conventional hemodialysis: pitfalls and lessons [published online ahead of print April 20, 2017]. Hemodial Int. doi:10.1111/hdi.12559. 28425578

[14] Liberati A Altman DG Tetzlaff J et al The PRISMA statement for reporting systematic reviews and meta-analyses of studies that evaluate healthcare interventions: explanation and elaboration. BMJ. 2009;339:b2700. 19622552 10.1136/bmj.b2700 PMC2714672

[15] Guyatt GH Oxman AD Vist G et al GRADE guidelines: 4. Rating the quality of evidence—study limitations (risk of bias). J Clin Epidemiol. 2011;64:407-415. 21247734 10.1016/j.jclinepi.2010.07.017

[16] Guyatt GH Oxman AD Kunz R et al GRADE guidelines: 8. Rating the quality of evidence—indirectness. J Clin Epidemiol. 2011;64:1303-1310. 21802903 10.1016/j.jclinepi.2011.04.014

[17] Guyatt GH Oxman AD Kunz R et al GRADE guidelines: 7. Rating the quality of evidence—inconsistency. J Clin Epidemiol. 2011;64:1294-1302. 21803546 10.1016/j.jclinepi.2011.03.017

[18] Guyatt GH Oxman AD Kunz R et al GRADE guidelines 6. Rating the quality of evidence—imprecision. J Clin Epidemiol. 2011;64:1283-1293. 21839614 10.1016/j.jclinepi.2011.01.012

[19] Guyatt GH Oxman AD Montori V et al GRADE guidelines: 5. Rating the quality of evidence—publication bias. J Clin Epidemiol. 2011;64:1277-1282. 21802904 10.1016/j.jclinepi.2011.01.011

[20] Higgins JP Altman DG Gotzsche PC et al The Cochrane Collaboration’s tool for assessing risk of bias in randomised trials. BMJ. 2011;343:d5928. 22008217 10.1136/bmj.d5928 PMC3196245

[21] DistillerSR. DistillerSR. 2013; http://systematic1review.net.

[22] The Cochrane Handbook for Systematic Reviews of Interventions Version 5.1.0. The Cochrane Collaboration, 2011 www.handbook.cochrane.org. Updated March 2011.

[23] Johansen KL Zhang R Huang Y et al Survival and hospitalization among patients using nocturnal and short daily compared to conventional hemodialysis: a USRDS study. Kidney Int. 2009;76:984-990. 19692997 10.1038/ki.2009.291 PMC5844181

[24] Lacson E Jr Xu J Suri RS et al Survival with three-times weekly in-center nocturnal versus conventional hemodialysis. J Am Soc Nephrol. 2012;23:687-695. 22362905 10.1681/ASN.2011070674 PMC3312497

[25] Chertow GM Levin NW Beck GJ et al Long-term effects of frequent in-center hemodialysis. J Am Soc Nephrol. 2016;27:1830-1836. 26467779 10.1681/ASN.2015040426 PMC4884113

[26] Marshall MR van der Schrieck N Lilley D et al Independent community house hemodialysis as a novel dialysis setting: an observational cohort study. Am J Kidney Dis. 2013;61:598-607. 23219810 10.1053/j.ajkd.2012.10.020

[27] Weinhandl ED Liu J Gilbertson DT Arneson TJ Collins AJ. Survival in daily home hemodialysis and matched thrice-weekly in-center hemodialysis patients. J Am Soc Nephrol. 2012;23:895-904. 22362906 10.1681/ASN.2011080761 PMC3338294

[28] Rocco MV Daugirdas JT Greene T et al Long-term effects of frequent nocturnal hemodialysis on mortality: the Frequent Hemodialysis Network (FHN) nocturnal trial. Am J Kidney Dis. 2015;66:459-468. 25863828 10.1053/j.ajkd.2015.02.331 PMC4549208

[29] Nesrallah GE Lindsay RM Cuerden MS et al Intensive hemodialysis associates with improved survival compared with conventional hemodialysis. J Am Soc Nephrol. 2012;23:696-705. 22362910 10.1681/ASN.2011070676 PMC3312510

[30] Ok E Duman S Asci G et al Comparison of 4- and 8-h dialysis sessions in thrice-weekly in-centre haemodialysis: a prospective, case-controlled study. Nephrol Dial Transplant. 2011;26:1287-1296. 21148270 10.1093/ndt/gfq724

[31] von Gersdorff G Schaller M Benzing T Barth C Outcomes of nocturnal hemodialysis in a large case-matched cohort. NDT Plus. ERA-EDTA Congress, Munich, 2010

[32] Kjellstrand CM Buoncristiani U Ting G et al Short daily haemodialysis: survival in 415 patients treated for 1006 patient-years. Nephrol Dial Transplant. 2008;23:3283-3289. 18458034 10.1093/ndt/gfn210

[33] Blagg CR Kjellstrand CM Ting GO Young BA. Comparison of survival between short-daily hemodialysis and conventional hemodialysis using the standardized mortality ratio. Hemodial Int. 2006;10:371-374. 17014514 10.1111/j.1542-4758.2006.00132.x

[34] Lockridge RS Kjellstrand CM. Nightly home hemodialysis: outcome and factors associated with survival. Hemodial Int. 2011;15:211-218. 21435157 10.1111/j.1542-4758.2011.00542.x

[35] Suri RS Lindsay RM Bieber BA et al A multinational cohort study of in-center daily hemodialysis and patient survival. Kidney Int. 2013;83:300-307. 22971996 10.1038/ki.2012.329

[36] Lindsay RM Leitch R Heidenheim AP Kortas C; London Daily/Nocturnal Hemodialysis Study. The London Daily/Nocturnal Hemodialysis Study—study design, morbidity, and mortality results. Am J Kidney Dis. 2003;42:5-12. 12830437 10.1016/s0272-6386(03)00531-6

[37] Van Eps CL Jones M Ng T et al The impact of extended-hours home hemodialysis and buttonhole cannulation technique on hospitalization rates for septic events related to dialysis access. Hemodial Int. 2010;14:451-463. 20955279 10.1111/j.1542-4758.2010.00463.x

[38] Bergman A Fenton SSA Richardson RMA Chan CT. Reduction in cardiovascular related hospitalization with nocturnal home hemodialysis. Clin Nephrol. 2008;69:33-39. 18218314 10.5414/cnp69033

[39] Zimbudzi E Samlero R. How do hospitalization patterns of home hemodialysis patients compare with a reasonably well dialysis patient cohort? Int J Nephrol Renovasc Dis. 2014;7:203-207. 24940077 10.2147/IJNRD.S65385 PMC4051731

[40] Lacson E Jr Wang W Lester K Ofsthun N Lazarus JM Hakim RM. Outcomes associated with in-center nocturnal hemodialysis from a large multicenter program. Clin J Am Soc Nephrol. 2010;5:220-226. 19965529 10.2215/CJN.06070809 PMC2827598

[41] Weinhandl ED Nieman KM Gilbertson DT Collins AJ. Hospitalization in daily home hemodialysis and matched thrice-weekly in-center hemodialysis patients. Am J Kidney Dis. 2015;65:98-108. 25085647 10.1053/j.ajkd.2014.06.015

[42] Culleton BF Walsh M Klarenbach SW et al Effect of frequent nocturnal hemodialysis vs conventional hemodialysis on left ventricular mass and quality of life: a randomized controlled trial. JAMA. 2007;298:1291-1299. 17878421 10.1001/jama.298.11.1291

[43] Weinhandl ED Gilbertson DT Collins AJ. Mortality, hospitalization, and technique failure in daily home hemodialysis and matched peritoneal dialysis patients: a matched cohort study. Am J Kidney Dis. 2016;67:98-110. 26319755 10.1053/j.ajkd.2015.07.014

[44] Kumar VA Ledezma ML Idroos ML Burchette RJ Rasgon SA. Hospitalization rates in daily home hemodialysis versus peritoneal dialysis patients in the United States. Am J Kidney Dis. 2008;52:737-744. 18752877 10.1053/j.ajkd.2008.06.013

[45] Nesrallah GE Li L Suri RS. Comparative effectiveness of home dialysis therapies: a matched cohort study. Can J Kidney Health Dis. 2016;3:19. 27006781 10.1186/s40697-016-0105-x PMC4802626

[46] Lacson E Jr Xu J Suri RS et al Survival with three-times weekly in-center nocturnal versus conventional hemodialysis. J Am Soc Nephrol. 2012;23:687-695. 22362905 10.1681/ASN.2011070674 PMC3312497

[47] USRDS 2014 Hospitalizations. 2015 (Accessed June 12, 2015, 2015, at http://www.usrds.org/2014/view/v2_04.aspx.

[48] Obi Y Streja E Rhee CM et al Incremental hemodialysis, residual kidney function, and mortality risk in incident dialysis patients: A cohort study. Am J Kidney Dis. 2016;68:256-265. 26867814 10.1053/j.ajkd.2016.01.008 PMC4969165

[49] Daugirdas JT Greene T Rocco MV et al Effect of frequent hemodialysis on residual kidney function. Kidney Int. 2013;83:949-958. 23344474 10.1038/ki.2012.457 PMC3855839

[50] Tsay SL. Self-efficacy training for patients with end-stage renal disease. J Adv Nurs. 2003;43:370-375. 12887355 10.1046/j.1365-2648.2003.02725.x

[51] Aliasgharpour M Shomali M Moghaddam MZ Faghihzadeh S. Effect of a self-efficacy promotion training programme on the body weight changes in patients undergoing haemodialysis. J Ren Care. 2012;38:155-161. 22429325 10.1111/j.1755-6686.2012.00305.x

[52] Suri RS Larive B Sherer S et al Risk of vascular access complications with frequent hemodialysis. J Am Soc Nephrol. 2013;24:498-505. 23393319 10.1681/ASN.2012060595 PMC3582201

[53] Macrae JM Ahmed SB Hemmelgarn BR; Alberta Kidney Disease Network. Arteriovenous fistula survival and needling technique: long-term results from a randomized buttonhole trial. Am J Kidney Dis. 2014;63:636-642. 24239019 10.1053/j.ajkd.2013.09.015

[54] Kotanko P Garg AX Depner T et al Effects of frequent hemodialysis on blood pressure: Results from the randomized frequent hemodialysis network trials. Hemodial Int. 2015;19:386-401. 25560227 10.1111/hdi.12255 PMC4490029

[55] Huang Z Gao D Letteri JJ Clark WR. Blood-membrane interactions during dialysis. Semin Dial. 2009;22:623-628. 20017832 10.1111/j.1525-139X.2009.00658.x

[56] Parekh RS Plantinga LC Kao WH et al The association of sudden cardiac death with inflammation and other traditional risk factors. Kidney Int. 2008;74:1335-1342. 18769368 10.1038/ki.2008.449

[57] Zimmermann J Herrlinger S Pruy A Metzger T Wanner C. Inflammation enhances cardiovascular risk and mortality in hemodialysis patients. Kidney Int. 1999;55:648-658. 9987089 10.1046/j.1523-1755.1999.00273.x

[58] Andrews J Guyatt G Oxman AD et al GRADE guidelines: 14. Going from evidence to recommendations: the significance and presentation of recommendations. J Clin Epidemiol. 2013;66:719-725. 23312392 10.1016/j.jclinepi.2012.03.013

[59] Covic A Bammens B Lobbedez T et al Educating end-stage renal disease patients on dialysis modality selection: clinical advice from the European Renal Best Practice (ERBP) Advisory Board. Nephrol Dial Transplant. 2010;25:1757-1759. 20392704 10.1093/ndt/gfq206

[60] Montori VM LeBlanc A Buchholz A et al Basing information on comprehensive, critically appraised, and up-to-date syntheses of the scientific evidence: a quality dimension of the International Patient Decision Aid Standards. BMC Med Inform Decis Mak. 2013;13(suppl 2):S5. 10.1186/1472-6947-13-S2-S5 PMC4044946 24625191

